# Supplementary material for: The impact of short-term exposure to near shore stressors on the early life stages of the reef building coral Montipora capitata
Source: PeerJ. 2020 Jul 3;8:e9415. doi: 10.7717/peerj.9415 (PMC7337034; doi:10.7717/peerj.9415)
Supplement: Table S2 — Each treatment was compared to the control with ambient conditions using one-way ANOVA with Dunnett’s Method. Statistical analysis was not done for settlement results due to low sample size. *Statistically significant (ANOVA P-value ≤ 0.05). [file peerj-08-9415-s002.docx]

Summary of results and statistics for fertilization, larval survival, and settlement. Each treatment was compared to the control with ambient conditions using one-way ANOVA with Dunnett’s Method. Statistical analysis was not done for settlement results due to low sample size. *Statistically significant (ANOVA P-value ≤ 0.05)

|  | Sediment | | Salinity | Temperature | Control |
| --- | --- | --- | --- | --- | --- |
|  | *100 mg l^-1^* | *200 mg l^-1^* | *28 ‰* | *31 °C* | *Ambient* |
| Fertilization (%) | 68.7 ± 6.3  F = 1.1  p = 0.3 | 77.8 ± 4.05  F = 1.1  p = 0.3 | 52.2 ± 5.9  F = 3.0  p = 0.08 | 50.0 ± 6.6  F = 3.5  p = 0.06 | 66.8 ± 5.7 |
| Larval survival (%) | 11.6 ± 5.2*  F = 13.9, p<0.0001 | 24.47 ± 5.5*  F = 13.9, p<0.0001 | 1.14 ± 6.1*  F = 38.8, p<0.0001 | 36.4 ± 8.6*  F = 4.9  p = 0.03 | 67.3 ± 10.7 |
| Settlement success (%) | 39.5 ± 14.3 | 20.0 ± 6.1 | --- | 25.3 ± 8.3 | 35.8 ± 6.0 |
